# Nanoparticle-induced intraperitoneal hyperthermia and targeted photoablation in treating ovarian cancer

**DOI:** 10.18632/oncotarget.4766

**Published:** 2015-08-03

**Authors:** Chao-Chih Wu, Yuh-Cheng Yang, Yun-Ting Hsu, T.-C. Wu, Chien-Fu Hung, Jung-Tang Huang, Chih-Long Chang

**Affiliations:** ^1^ Graduate Institute of Mechanical and Electrical Engineering, National Taipei University of Technology, Taipei City, Taiwan; ^2^ Department of Obstetrics and Gynecology, Mackay Memorial Hospital, Taipei City, Taiwan; ^3^ Department of Medical Research, Mackay Memorial Hospital, Taipei City, Taiwan; ^4^ Department of Medicine, Mackay Medical College, Sanchi, New Taipei City, Taiwan; ^5^ Department of Pathology, The Johns Hopkins University, Baltimore, Maryland, USA; ^6^ Department of Oncology, The Johns Hopkins University, Baltimore, Maryland, USA; ^7^ Department of Obstetrics and Gynecology, The Johns Hopkins University, Baltimore, Maryland, USA; ^8^ Department of Molecular Microbiology and Immunology, The Johns Hopkins University, Baltimore, Maryland, USA

**Keywords:** hypethermia, gold nanoshells, ovarian cancer

## Abstract

Hyperthermic intraperitoneal chemotherapy is effective in treating various intra-abdominal malignancies. However, this therapeutic modality can only be performed during surgical operations and cannot be used repeatedly. We propose repeatedly noninvasive hyperthermia mediated by pegylated silica-core gold nanoshells (pSGNs) *in vivo* with external near-infrared (NIR) laser irradiation. This study demonstrated that repeated photothermal treatment can effectively eliminate intraperitoneal tumors in mouse ovarian cancer models without damage of normal tissues. By conjugating pSGNs with anti-human CD47 monoclonal antibody, a significant photoablative effect can be achieved using lower amount of pSGNs and shorter NIR laser irradiation. Conjugated pSGNs specifically targeted and bound to cancer cells inside the peritoneal cavity. Our results indicate the possibility of a noninvasive method of repeated hyperthermia and photoablative therapies using nanoparticles. This has substantial clinical potential in treating ovarian and other intraperitoneal cancers.

## INTRODUCTION

Epithelial ovarian cancer is the most lethal gynecological malignancy worldwide [1]. Most cases of ovarian cancer are not diagnosed until the advanced stage, because it is generally symptomless and lacks reliable biomarkers in the early-staged diseases [2–4]. The standard primary treatment for this malignancy is an optimal debulking operation followed by platinum-based chemotherapy [5]. Although the initial adjuvant chemotherapy is mostly successful, most patients experience repeated remission and relapse with gradually decreasing intervals and eventually develop drug resistance [6]. Thus, new therapeutic modalities are urgently required for combating this fatal disease.

Ovarian cancer is considered a disease of the peritoneal cavity because of the nature of its occurrence and spread. Several intraperitoneal treatment modalities have been advocated for treating residual or microscopic tumors after debulking surgery [7]. For example, hyperthermic intraperitoneal chemotherapy (HIPEC) has become a frequently adopted adjuvant therapy after cytoreductive surgery in the treatment of ovarian cancer [8, 9]. Numerous hypotheses have been proposed as the mechanism of this hyperthermic treatment, such as direct thermal cytotoxicity and decreased blood perfusion in cancer lesions, creating an acidic, hypoxic, and nutritionally deprived microenvironment [10]. Hyperthermia and thermal chemosentization, are thought to be able to increase the tissue perfusion and tumor permeability of cytotoxic agents [11]. Hyperthermia is assumed to cause tumor-associated immune responses through the induction of surface proteins. However, hypothermia also triggers a powerful resistance mechanism in cancer cells, which is mediated by the induction of heat shock proteins (HSPs). HSPs rapidly repair the thermal damage of proteins, leading to thermoresistance, which contributes to the survival of cancer cells.

Although HIPEC is effective in treating ovarian and other intraperitoneal cancers, hyperthermia induced by circulating heated fluid can only be administered during surgical operations and can hardly be applied repeatedly. Therefore, its effectiveness is limited to one-time use and is nonspecific. Thus, a target-specific strategy that can be used repeatedly must be developed. Recently, a strategy involving the generation of heat by various nanoparticles using out-source energy has been reported and tested in cancer treatment [12]. Among the used nanoparticles, pegylated silica-core gold nanoshells (pSGNs) were developed through a process involving adsorbing laser energy at a specific range of wave lengths to induce surface plasma resonance (SPR) and subsequent heat generation [13]. In this study, we attempted to induce internal heat generation using pSGNs. Tissue penetration by using various wave lengths of energy-delivering laser beams to induce heat generation of pSGNs inside the peritoneal body has been tested. The wave lengths located in the near-infrared (NIR) region (750 to 850 nm), called the NIR window, have the lowest capacity for adsorption by water and hemoglobin and enable the deepest tissue penetration [14]. By adjusting the ratio of the silica core diameter and the thickness of the gold nanoshell, the maximal adsorption wave length can be tuned to between 750 and 850 nm for hyperthermic therapy. Because this type of energy delivery is noninvasive, this therapy can be performed repeatedly to achieve the maximal therapeutic effect. Furthermore, gold nanoshells can be conjugated to biomolecules through simple surface modification [15]. By conjugating with antibodies that target tumor surface molecules, gold nanoshells administered into the peritoneal cavity can specifically attach to tumor cells, enabling target-specific photoablative therapy through extracorporeal NIR laser irradiation.

Photothermal therapy mediated by an NIR laser to trigger the surface plasma resonance of gold nanoshells has been demonstrated to be effective in cancer treatment on several subcutaneous tumor models [16–19]. On the basis of this strategy, we developed a noninvasive photothermal application to perform repeated peritoneal hyperthermia and achieve significant therapeutic effect in both murine and human i.p. xenograft ovarian cancer models. CD47 has originally been reported to be overexpressed on the cell surface of ovarian cancer and investigated as a potentially broad cancer therapy target [20, 21]. Therefore, the gold nanoshells were conjugated with an anti-CD47 antibody to specifically target cells with CD47 overexpression. These pSGNs were determined to be internalized into cells expressing surface CD47 but no other cells. When applied *in vivo* with an external NIR laser, the direct photoablative effect on engulfed tumor cells reduced the required amount of NIR and target-specific pSGNs to achieve a similar therapeutic effect. This noninvasive, repeatedly applicable photothermal therapy that induces hyperthermia or targeted photoablation provides a proof of concept and constitutes a substantial strategy in treating i.p. tumors.

## RESULTS

### Characteristics of pSGNs and their photothermal effect *in vitro*

**Figure 1:**
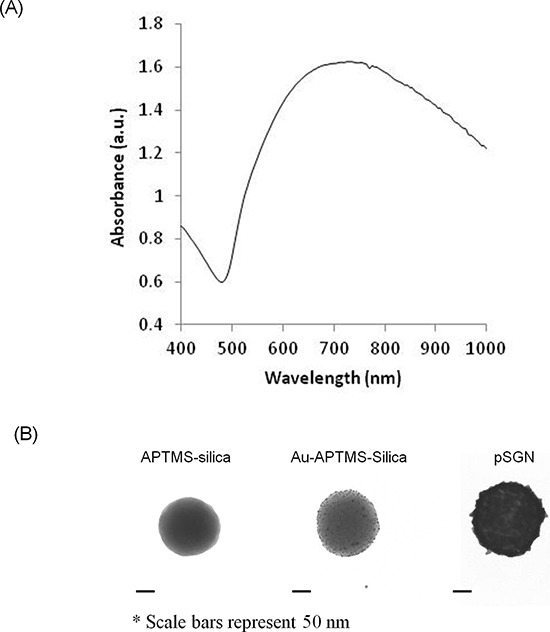
Characterization of SGNs **A.** Absorbent spectrum scanning of silica-core gold nanoshells. The peak of absorbent spectrum was approximately 750 nm. **B.** TEM images of APTMS-functionalized silica core, Au-APTMS-silica, and silica-core gold nanoshell. The size of the scale bar was 50 nm.

The results demonstrated that the pSGNs fabricated in this study exhibited the maximal plasma absorption spectrum in the NIR range (approximately 750 nm; Figure 1A). Morphologies of the silica cores, gold nanoparticle-seeded silica cores, and pSGNs examined under transmission electron microscope (TEM) are shown in Figure 1B. The TEM images of 50 pSGNs nanoparticles determined using ImageJ software show that the average diameter of the pSGNs was 156.41 ± 15.38 nm. The concentration of pSGNs was measured using a scanning electron microscope (SEM) to calculate the ratio of nanoparticles with the known concentration of 3-μm latex beads, and analyzed using 10 independent images generated by ImageJ. When the absorption of pSGNs was adjusted to OD_800_ = 6, the concentration was approximately 1 × 10^10^/mL (1.09 × 10^10^ ± 2.2 × 10^9^).

**Figure 2:**
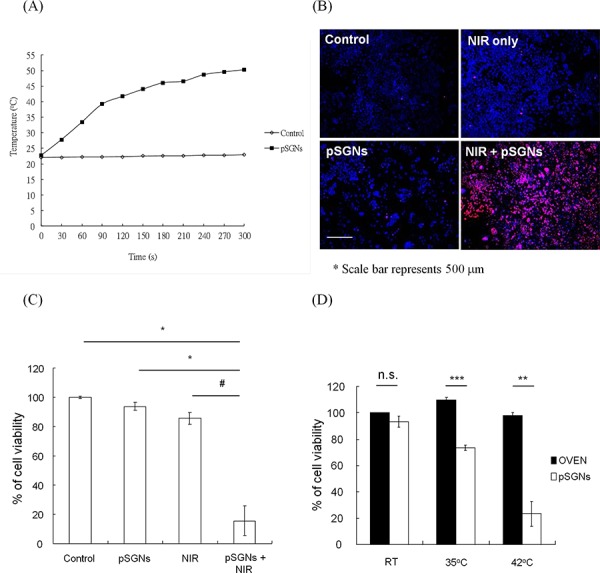
Temperature increase of pSGNs induced by surface plasmon resonance (SPR) caused by 808-nm NIR irradiation **A.** Temperature elevation curve of 1 mL of pSGNs-containing solution with culture medium (ratio 3:1, OD_800_ = 6) irradiated using a 2000-mW 808-nm NIR laser. The temperature change was monitored every 30 s using a thermocouple. The culture medium with pSGNs absorbed the energy of the NIR laser and caused the temperature to increase to nearly 30°C in 5 min, but the medium-only control did not. **B.** 1 × 10^5^ mouse ovarian cancer ID8 cells were seeded into a 24-well plate and double-stained with Hochest 33342 and propidium iodide 18 h after NIR laser irradiation. Blue represents Hochest 33342 cell nuclei; red represents propidium iodide-stained dead cell nuclei. Cells that did not receive any treatment, only received NIR laser irradiation, and those that were only cocultured with pSGNs did not exhibit death. However, cell death was significant in the group treated with pSGNs and NIR laser irradiation. The size of the scale bar was 500 μm. **C.** Cell viability 18 h after NIR laser irradiation. The irradiation duration was set to when the medium temperature of the NIR + pSGNs cohort reached 42°C. The cell viability was determined using an MTT assay. Compared with all other groups, the cells that were cultured in pSGNs-containing medium and irradiated with the NIR laser exhibited significant cell death. **D.** Cell viability 18 h after the cultured cells were heat-treated in an oven for 10 min and with an NIR laser + pSGNs, achieving the same temperature. The viability for both groups was not significantly different at room temperature. However, the viability of cells treated with photothermal therapy was lower than that of cells heated using an oven at 35 and 42°C. These results demonstrated that heat generated with an NIR laser induced an SPR effect of pSGNs that can kill tumor cells *in vitro*. The culture medium heated in an oven to the same temperature did not exhibit significant cell death, implying that the local temperature surrounding the pSGNs stimulated by the NIR laser irradiation may be higher than that measured by the thermocouple and could effectively kill proximal tumor cells. The error bar in each chart represents the standard error. (*P* < 0.05#, <0.01*, <0.001**)

To evaluate whether the fabricated pSGNs were able to absorb the 808-nm NIR laser and generate heat for photothermal therapy, the pSGNs were adjusted to OD_800_ = 6 in 10% trehalose and mixed in cell culture medium at a ratio of 1:3. After a session of NIR laser irradiation (2 W, 808 nm), the temperature curve of the 1-mL pSGNs/culture medium mixture was measured using the thermocouple every 30 s. Figure 2A shows the temperature rising curve of the mixture within 5 min of NIR laser irradiation. The temperature increase in the medium mixed with pSGNs almost reached 30°C (from 22.7 to 50.3°C) after 5 min of NIR irradiation. However, the temperature change in the control medium without pSGNs was only approximately 1°C (22 to 23°C) during NIR irradiation. These results demonstrated that the 808-nm NIR laser can induce SPR of the fabricated pSGNs and generate heat, increasing the temperature of the cell culture medium.

To further evaluate the tumoricidal ability of photothermal therapy induced by SPR of fabricated pSGNs excited with NIR laser, ID8 cells were cultured in 1 mL of culture medium mixed with gold nanoshells, as previously described, in a 24-well plate and irradiated with an NIR laser for 5 min. On the next day, the nucleated cells were stained with Hochest 33342, and dead cells were stained with propidium iodide. In the control, NIR-only, and pSGNs-only groups, few and no cell deaths were observed. However, the group treated with pSGNs and irradiated with an NIR laser clearly exhibited cell death (Figure 2B). These results demonstrated that the prepared pSGNs can generate heat through the SPR effect and cause cell death *in vitro*.

Clinically standard i.p. hyperthermia in the treatment of ovarian cancer is generally performed at 41 to 43°C. Hence, in this study, the cell viability was confirmed when the temperature of the culture medium was increased to 42°C through the photothermal effect *in vitro*. Figure 2C shows the viability of ID8 cells 18 h after NIR laser irradiation. The viability of the NIR + pSGNs group was significantly reduced compared with that of the nontreated control group (*P* = 0.006, pSGNs + NIR vs. control), pSGNs-only (nanoshell) group (*P* = 0.0083, pSGNs + NIR vs. pSGNs), and NIR-only group (*P* = 0.0111, pSGNs + NIR vs. NIR). No difference in the cell viability of the pSGNs-only, NIR-only, and control groups was determined. The thermoablative effect of directly heating the medium by using an oven (environmental heating) was compared with that of heating the medium through photothermal treatment. No difference in the cell viabilities was observed regardless of whether the cells were incubated at room temperature, 35°C, or 42°C in an oven. However, cell viabilities decreased to 73% at 35°C and 23% at 42°C when heated through photothermal treatment with pSGNs and NIR laser irradiation (Figure 2D). To preclude the cytotoxic effect of pSGNs on ID8 cells, various concentrations of pSGNs were cocultured with ID8 cells for 48 h, and only mild growth inhibition was observed at twice the concentration of pSGNs (5 × 10^9^/ mL) that we used for photothermal therapy (Supplementary Figure 1). This result implied that pSGNs after NIR laser excitation likely acted as hot cores, with a higher temperature than that of the medium recorded by the thermocouple, and were capable of killing proximal cancer cells.

### Near-infrared laser abdominal wall penetration test

**Figure 3:**
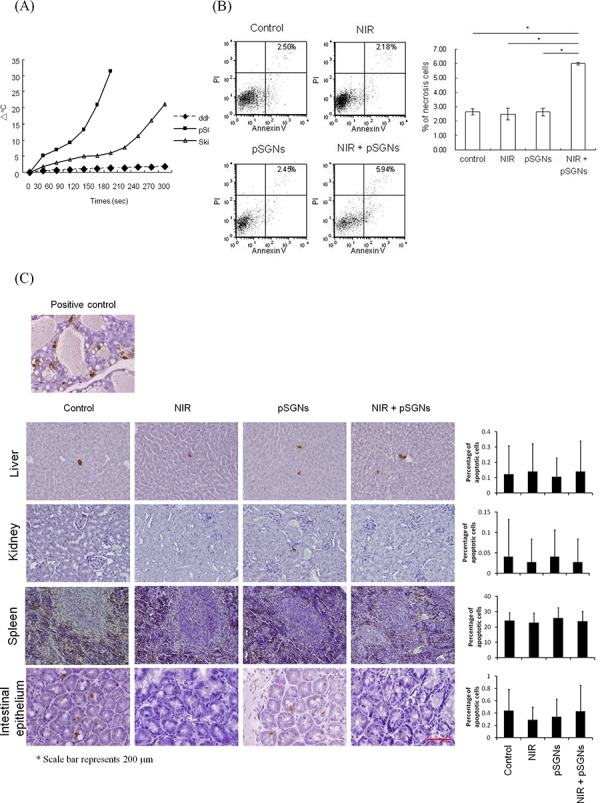
NIR laser can penetrate the mouse abdominal wall and cause an SPR effect of pSGNs to kill cancer cells *in vivo* **A.** The abdominal skin of NOD/SCID mice was dissected and placed on a glass slide covering the 96-well plate. The abdominal skin was irradiated using a 2000-mW NIR laser, and the temperature of 100 μL of pSGNs (OD_800_ = 6) in the 96-well plate under the abdomen wall was monitored using a thermocouple. The wells that contained distilled water or pSGNs and were irradiated directly with the NIR laser without a mouse abdominal skin covering were used as control. The pSGNs under the mouse abdominal skin that were irradiated with the NIR laser generated heat and increased the solution temperature. These results imply that the NIR laser can penetrate the NOD/SCID mouse abdomen and cause pSGNs to generate heat through the SPR effect. **B.** 1 × 10^6^ ID8 cells were intraperitoneally injected into NOD/SCID mice. After 3 days, the mice were divided into four groups of five mice and intraperitoneally injected with 2 mL of pSGNs (OD_800_ = 1.5) or 10% trehalose and irradiated with an 808-nm NIR laser (3.2 W/cm^2^) in five different areas on the abdomen for a total of 5 min. One day after the final NIR irradiation, the mice implanted with ID8 cells were sacrificed, and i.p. lavage was performed to collect cancer cells. The lavaged cells were then stained with annexin V and propidium iodide. GFP-positive cancer cells were gated, and annexin V-PI double-positive necrosis cells were analyzed using flow cytometry. No significant difference in the percentage of necrosis in i.p. cancer cells was observed among control, NIR-laser irradiated, and i.p. pSGNs-only groups. However, the percentage of necrosis in cancer cells was significantly increased in the groups that received i.p. photothermal treatment mediated by pSGNs. **C.** The normal tissues from the mice of each group were dissected and fixed in 10% formalin. The damage of normal tissues in the intraperitoneal cavity was evaluated using a TUNEL assay, and the percentage of damaged cells was determined using ImageJ software. Normal female rodent mammary gland tissue, 3 to 5 days after weaning of rat pups, was used as positive control. The size of the scale bar was 200 μm. This result demonstrated that photothermal therapy did not cause noticeable damage in normal tissues *in vivo*. The error bar represents the standard error. (*P* < 0.01*)

To treat the peritoneal tumors of mice, an 808-nm NIR laser must penetrate the mouse abdominal wall and generate an SPR response from the pSGNs inside the peritoneal cavity. To mimic this condition, pSGNs-containing solution covered by a layer of mouse abdominal skin was irradiated using an NIR laser. The temperature of the pSGNs-containing solution increased gradually as the duration of the irradiation increased (Figure 3A). This result demonstrated that the 808-nm NIR laser can penetrate the mouse abdominal skin and cause the SPR of pSGNs to generate heat, although this is not as efficient as direct irradiation.

This study further investigated whether this mode of photothermal therapy can kill ovarian cancer cells *in vivo*. First, NOD-SCID mice were treated with 808 nm of NIR laser irradiation in five areas on the abdomen for various durations at 3.2 W/cm^2^ using a 0.54-cm^2^ laser spot to evaluate the damage on the skin caused by the NIR laser. On the next day, no injury was observed on the abdomen of the mice (Supplementary Figure 2). Subsequently, mice exhibiting i.p. ID8 (expressing green fluorescence, GFP) ovarian tumor cells were administered with pSGNs and irradiated using an 808-nm NIR laser. On the next day, peritoneal cells were lavaged out, stained with annexin V and PI and analyzed using flow cytometry. To determine whether normal tissues were damaged after the hyperthermia induced through photothermal therapy, liver, spleen, kidney, and bowel tissues of the irradiated mice were dissected and embedded in paraffin for an in situ apoptosis assay and Ki-67 staining. The ID8 cells were distinguished from the host cells by gating the GFP expressing cells and analyzed. Flow data showed that the number of annexin V/PI double-positive cancer cells in the group that received photothermal therapy was twice as high as that of the other groups (Figure 3B; *P* = 0.0024, NIR + pSGNs vs. control; *P* = 0.007, NIR + pSGNs vs. NIR only; *P* = 0.0034, NIR + pSGNs vs. pSGNs only). In addition, on the basis of a TUNEL assay and Ki-67 staining, no cell damage in the intraperitoneal vital organs was observed (Figure 3C and Supplementary Figure 3). This result showed that the NIR laser penetrated the abdominal wall, exerted a photothermal effect (i.p. hyperthermia), and caused tumor cell death without harming normal intraperitoneal tissues.

### Repeated application of photothermal therapy for intraperitoneal ovarian cancer

**Figure 4:**
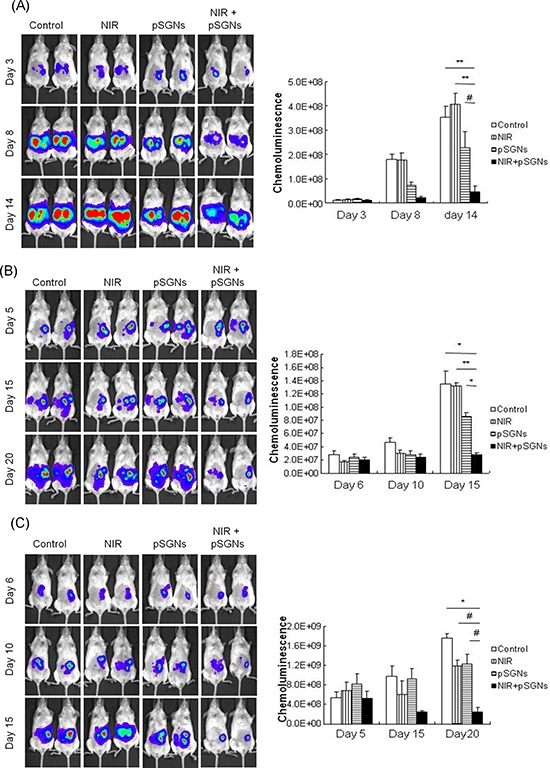
Intraperitoneal photothermal therapy induced by pSGNs with NIR laser irradiation **A.** Three hundred thousand ID8 cells, **B.** one million TOV21G cells and **C.** 1 × 10^6^ SKOV-3 cells were intraperitoneally injected into NOD/SCID mice. After 2 days for ID8 cells and after 6 days for TOV21G and SKOV-3 cells, the mice were intraperitoneally injected with 2 mL of pSGNs (OD_800_ = 1.5) and irradiated using an 808-nm NIR laser (3.2 W/cm^2^) in five areas on the abdomen for a total of 5 min. The NIR laser irradiation was repeated every 3 to 4 days, and the pSGNs were supplemented on the third and eighth days. Tumor sizes were represented by the chemoluminescent intensity and determined using an IVIS spectrum system. In these tumor cell models, the tumor growth was slightly inhibited through the NIR laser irradiation or the pSGNs treatment. However, intraperitoneally administered pSGNs combined with NIR laser irradiation significantly inhibited tumor growth compared with the control, NIR-only, and pSGNs-only groups in both tumor cell models. These results demonstrated that repeated photothermal therapy can inhibit i.p. tumor growth *in vivo*. The error bars in each chart represent the standard error. (*P* < 0.05#, <0.01*, <0.001**, <0.0001***)

Because the photothermal effect can kill cancer cells *in vitro* and penetrate skin, generating hyperthermia intraperitoneally, we investigated whether this treatment modality can be used to treat i.p. ovarian cancer *in vivo*. Mouse ovarian cancer cell ID8 and human ovarian cancer cell TOV21G and SKOV-3 were intraperitoneally grown inside immunodeficient NOD/SCID mice. After multiple treatments of pSGNs combined with NIR laser irradiation, the mice exhibited tumor growth inhibition. However, both NIR-only and pSGNs-only cohorts exhibited little and no tumor growth inhibition (In ID8 model, *P* = 0.0009, NIR + pSGNs vs. control; *P* = 0.0003, NIR + pSGNs vs. NIR only; *P* = 0.0408, NIR + pSGNs vs. pSGNs only; in TOV21G model, *P* = 0.0024, NIR + pSGNs vs. control; *P* = 0.0003, NIR + pSGNs vs. NIR only; *P* = 0.0018, NIR + pSGNs vs. pSGNs only; in SKOV-3 model, *P* = 0.0019, NIR + pSGNs vs. control; *P* = 0.0275, NIR + pSGNs vs. NIR only; *P* = 0.0325, NIR + pSGNs vs. pSGNs only; Figure 4). This result demonstrated the therapeutic efficacy in treating i.p. ovarian cancer by using pSGNs and NIR laser irradiation *in vivo* and implies that the noninvasive photothermal strategy has potential in clinical application.

### Characterization of targeted pegylated silica-core gold nanoshells

**Figure 5:**
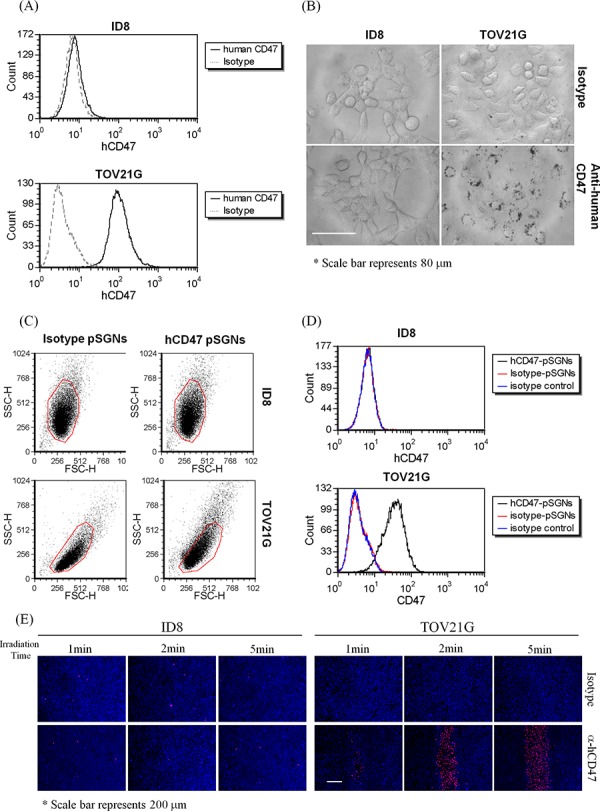
Specific cell binding of anti-human CD47 antibody-conjugated pSGNs **A.** Expression of human CD47 on ID8 and TOV21G cells. Both cells were stained with fluorescent anti-human CD47 antibodies and analyzed using flow cytometry. TOV21G cells strongly expressed human CD47, whereas ID8 cells did not. **B.** The anti-human CD47 and isotype antibody-conjugated pSGNs (OD_800_ = 6) were mixed with cell culture medium at a ratio of 1:10 and added into the ID8 or TOV21G cell culture wells. The cells were cultured at 37°C overnight and washed twice with fresh medium. The cell images were recorded using a DIC microscope at 640× magnitude. The size of scale bar was 80 μm. **C, D.** pSGNs conjugated with isotype control antibody or anti-human CD47 antibody were mixed with ID8 cells and TOV21G cells. The side scatters and fluorescent shift of cells caused by particle binding on human CD47-positive TOV21G cells were determined through flow analysis. **E.** 1 × 10^5^ of ID8 cells and 2 × 10^5^ of TOV21G cells were seeded into 24-well plates and cultured overnight at 37°C. The antibody-conjugated pSGNs (OD_800_ = 6) were mixed with cell culture medium at a ratio of 1:10, added into the ID8 or TOV21G cell culture wells, and cultured for 2 h. The cells were washed twice in new medium and then irradiated using a 2000-mW NIR laser on each well for 1, 2, and 5 min. All cells were stained with Hochest 33342 and propidium iodide 18 h after NIR laser irradiation. The fluorescence images of the cell staining were observed using a fluorescence microscope. These data from various optical analyses demonstrated that anti-human CD47 antibody-conjugated pSGNs can specifically bind to human TOV21G ovarian cancer cells, but not to mouse cells, and causes cell death through photothermal effects. The size of the scale bar was 200 μm.

Currently, the i.p. hyperthermia performed in HIPEC for treating ovarian cancer is typically performed immediately after a debulking operation. Using a targeted gold nanoshell in i.p. photothermal therapy is considerably more efficient in removing residual microscopic lesions if the nanoshell can be modified to be tumor-specific. Human ovarian cancer cell TOV21G expressing human CD47 on the cell surface and mouse ovarian cancer cell ID8 not expressing human CD47 was used as tumor models (Figure 5A). The pSGNs were first conjugated to anti-human CD47 antibodies (hCD47-pSGNs); the conjugated pSGNs recognized CD47 molecules expressed on the surface of human ovarian cancer cells TOV21G but not on mouse ovarian cells ID8. Under light microscopy, hCD47-pSGNs were observed to aggregate around TOV21G cells, indicating their binding to TOV21G cells. By contrast, isotype antibody-conjugated pSGNs did not bind to the cells and were washed out by fresh medium before observation (Figure 5B). However, both types of antibody-conjugated pSGNs did not bind to mouse ID8 cells and were washed out. This suggests that hCD47-pSGNs can bind to TOV21G cells and increase granulites, causing a shift of the side scatter signal. This phenomenon did not occur in mouse ID8 cells (Figure 5C). When stained with fluorescent secondary antibodies, the hCD47-pSGNs bound to TOV21G but not to ID8 cells (Figure 5D). These results confirmed that hCD47-pSGNs specifically bound to human TOV21G cells.

To examine if hCD47-pSGNs can target and destroy human cancer cells when subjected to NIR laser irradiation, hCD47-pSGNs were cocultured with TOV21G and ID8 cells. After NIR laser irradiation, the TOV21G cells cocultured with hCD47-pSGNs died in 18 h at the irradiated area, whereas ID8 cells were not affected (Figure 5E and Supplementary Figure 4). Hence, the targeted pSGNs conjugated withanti-CD47 antibodies are likely able to efficiently kill cancer cells in a direct photoablative manner.

### Photoablative therapy using targeted hCD47-pSGNs leads to the killing of i.p. ovarian cancer *in vivo*

**Figure 6:**
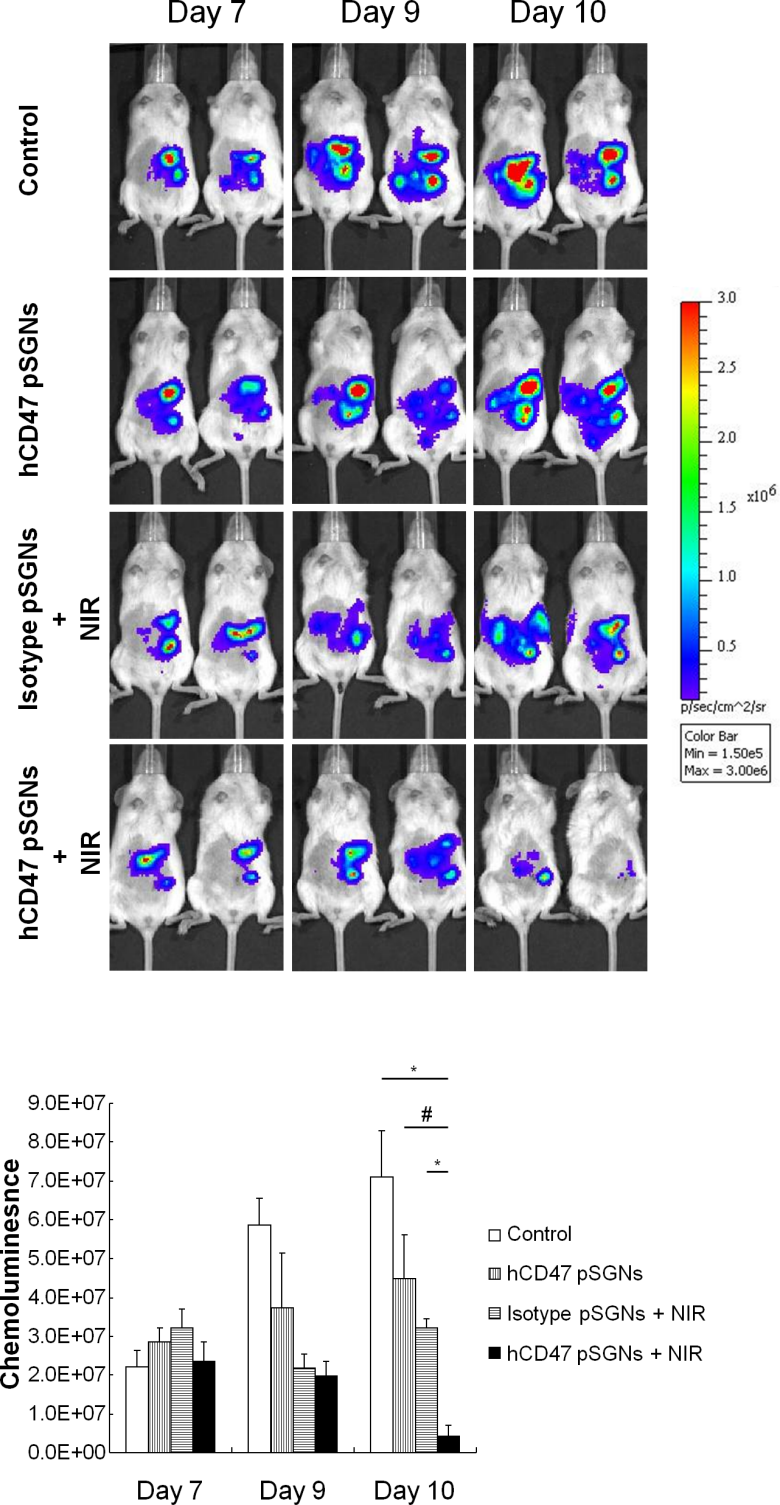
Target-specific intraperitoneal photosthermal therapy of human ovarian cancer One million TOV21G-luc cells were intraperitoneally injected into NOD/SCID mice. After 7 days, 2 mL of pSGNs conjugated with anti-human CD47 antibodies (OD_800_ = 0.625) or isotype pSGNs (OD_800_ = 1.5) were intraperitoneally injected into tumor-bearing mice. Mice that were intraperitoneally injected with 2 mL of 10% trehalose were used as controls. One day after pSGNs injection, the mice were irradiated using an 808-nm NIR laser (3.2 W/cm^2^) in five areas of the abdomen for 5 min. Tumor growth curves were represented by the chemoluminescent intensity and determined using an IVIS spectrum system. Compared with other groups, the group treated with anti-human CD47 conjugated pSGNs and NIR laser irradiation had the strongest therapeutic effect on the human ovarian cancer cell xenograft model. The error bar represents standard error. (*P* < 0.05#, <0.01*)

To evaluate the antitumor effect of targeted pSGNs *in vivo*, mice with a TOV21G tumor were intraperitoneally injected with hCD47-pSGNs (2 mL with OD_800_ = 0.625) and isotype-pSGNs (2 mL with OD_800_ = 1.5) and irradiated using an 808-nm NIR laser. After 2 days, the tumor sizes of the mice that received hCD47-pSGNs and NIR irradiation were substantially smaller than those of the nontreated control group (*P* = 0.0057, control vs. hCD47-pSGNs + NIR), hCD47-pSGNs without NIR group (*P* = 0.0244, hCD47-pSGNs vs. hCD47-pSGNs + NIR), and isotype-pSGNs with NIR group (*P* = 0.0018, pSGNs + NIR vs. hCD47-pSGN + NIR; Figure 6). Our data did show hCD47-pSGNs, but not isotype antibody conjugated pSGNs can bind to TOV21G cells *in vivo* (Supplementary Figure 5). This may help in enhancing the passive targeting of pSGNs (Supplementary Figure 6) to tumor tissues. These results demonstrated that targeted pSGNs enhanced tumor treatment through direct photoablation of tumor cells and achieved similar antitumor effect with less irradiation and a reduced amount of gold nanoshells. This targeted photoablative strategy may enhance tumor treatment and reduce possible side effects.

## DISCUSSION

Hyperthermia has been proven to be effective in cancer treatment and involves several documented mechanisms, including decreasing blood supply, acidifying tumor lesions, inhibiting RNA synthesis, and halting mitosis. Combining hyperthermia with chemotherapy constituted a superior therapeutic effect because of increased tissue perfusion and cell permeability of drugs. Our previous study indicated an immune-enhancing effect of hyperthermia when combining it with α-galactosylceramide in tumor-killing [22]. Regardless of the mechanisms, the effectiveness of hyperthermia in cancer therapy is generally related to the temperature and duration of the treatment. Abundant experimental and clinical studies have indicated that malignant cells are destroyed in the range of 41 to 43°C. However, because of the invasiveness of the procedure, i.p. hyperthermia is typically performed concomitantly immediately after a debulking operation. The antitumor effect of hyperthermia in this setting does not last long, and the tumor might eventually regrow. Therefore, whether repeated hyperthermia can enhance therapeutic effectiveness warrants further research. The development of a therapeutic modality that enables repeated noninvasive i.p. hyperthermia is therefore required for this concept to be feasible and applicable. This study adopted a noninvasive photothermal therapy that subjects gold nanoshells inside the peritoneal cavity to external NIR laser irradiation. Although previous studies have reported that the intensity of NIR laser decreased with the depth of tissue [23] and little evidence exists proving that an NIR laser can penetrate to the required depth to achieve sufficient heating throughout the peritoneal cavity, our data verifies that the NIR laser heated the gold nanoshells inside the peritoneal cavity and raised the temperature of the surrounding solution through a layer of mouse abdominal wall. The consequence of repeated photothermal therapy was a substantial antitumor effect without causing damage to the normal tissues. Regarding how efficiently the peritoneal cavity can be heated in a human, we speculate that this will be resolved through improvements of the light source or the use of alternative energy-generating technologies. Compared with conventional hyperthermia, which generates heat by filling the peritoneal cavity with hot solution, photothermal therapy generates heat through gold nanoshell particles. Each nanoparticle becomes a hot core, transmitting heat to the surrounding solution and increasing the solution's temperature. Consequently, the cultured cells near the gold nanoshells possibly had a higher temperature than was measured in the culture medium. This explains why heating the medium using photothermal therapy with pSGNs and the NIR laser caused substantially more cell death than heating it using an oven with the same temperature.

Because an NIR laser scatters when penetrating tissues, the energy delivered is generally considerably lower than that from direct irradiation. This was reflected by the temperature in the *in vitro* gold nanoshell solution covered with an abdominal layer that was lower than the temperature of the solution that underwent direct irradiation without cover. Nevertheless, gold nanoshells irradiated by a scattering NIR laser can still cause considerable tumor cell death through the hot-core mechanism. In general, normal tissues are more thermally resistant than tumor cells [24]. The hot core gold nanoshells irradiated by the scattering NIR laser in this therapy were likely not overheated, and therefore, did not damage normal tissues. Repeated NIR laser irradiation might augment the photothermal effect each time before the repair of damaged tumor cells and cause considerably more tumor cell death than the addition of tumor-killing effects through each single treatment.

Theoretically, the targeted delivery of gold nanoshells to a tumor lesion is considerably more effective in photothermal therapy. Antibody-conjugated gold nanoshells are expected to recognize and bind to tumor cells in the peritoneal cavity. Conjugated gold nanoshells with antibodies that target tumor-specific surface proteins or antigens may enhance therapeutic effects. In this study, CD47 was used because it has been identified as a tumor antigen of ovarian cancer. CD47 has been reported to be overexpressed on the surface of cancer cells and exhibit weak or no expression on normal cells [20, 25, 26]. Thus, anti-human CD47 antibody-conjugated pSGNs can specifically target ovarian cancer cells and enable effective photothermal therapy with a reduced amount of gold nanoshells. Blocking the surface of cells' CD47 with anti-CD47 antibodies has been reported to inhibit tumor growth by blocking the defense “do not eat me” signal of malignant cells, enabling macrophages to engulf the cancer cells [27, 28]. This might augment the presentation of tumor antigen through macrophages to T cells and induce tumor-specific immune responses. Thus, CD47-conjugated gold nanoshells exhibited considerably strong antitumor effects that not only damaged the cancer cells through direct photoablation but also triggered the host antitumor immunity.

In clinical settings, i.p. hyperthermia has always been combined with other modalities to treat peritoneal malignancies such as chemotherapy and radiation therapy. HIPEC entails an inherent safety concern regarding the contamination of medical staff with chemotherapeutic drugs in the operation room. Most importantly, the efficacy of HIPEC is usually limited because i.p. hyperthermia can only be applied once during an operation. In this study, a noninvasive nanoshell mediator was designed to generate heat in the peritoneal cavity, and the peritoneal hyperthermia was evoked repeatedly in mice using an NIR laser. In addition, various types of gold nanoshells were designed as carriers of cytotoxic agents. In doxorubicin-carrying poly(lactide-co-glycolide; PLGA) nanoparticles, the encapsulated drug can be modulated to be released when exposed to an extracorporeal NIR laser irradiation and trigger the degradation of PLGA particles [29]. Regarding the limited penetration depth of an NIR laser in human tissues that may not satisfy clinical requirements, alternative energy sources such as radio frequencies and microwaves have been tested to trigger the heat generation of gold nanoparticles in deep tissues efficiently [30–33]. However, because the residuals of tumor deposits are possibly obstructed by tissues and cannot interact with nanoparticles suspended in the intraperitoneal cavity, the optimal sizes of nanoparticles with specific targeting moleculars must be investigated for superior tumor tissue penetration, targeting, and clearance [34]. Furthermore, for various malignancies, treatments must be optimized according to the site of the tumor and its biological characteristics using appropriate energy sources and particle administered strategies. In summary, intraperitoneal gold nanoparticles irradiated using external energy sources are effective in treating diseases that are spread in the peritoneal cavity such as ovarian cancers. Gold nanoshells have also been reported to be safe *in vivo* [35–38]. Thus, the application of gold nanoshell-mediated i.p. hyperthermia and targeted thermoablation is clinically feasible.

In conclusion, this study demonstrated a noninvasive photothermal therapy involving gold nanoshells and NIR laser irradiation; the therapy enables repeated intraperitoneal hyperthermia and can effectively kill cancer cells in *vivo*. The gold nanoshells conjugated with anti-CD47 antibodies are highly efficient in killing cancer cells, and reduce the required amount of gold nanoshells and duration of NIR irradiation. We anticipate that additional energy sources and modified nanoparticles for drug delivery will be developed. Therefore, in the future, the proposed method can be applied in the clinical treatment of patients with peritoneal tumors.

## MATERIALS AND METHODS

### Mice and cell lines

NOD/SCID mice were purchased from BioLasco, Taiwan. The mice were reared in specific pathogen-free conditions. Mouse ovarian cancer cell line ID8-luc (C57BL/6 origin) and human ovarian cancer cell line TOV21G and SKOV-3 (ATCC) were cultured in RPMI 1640 medium (Gibco, Grand Island, NY, supplemented with 10% fetal bovine serum (FBS; Biological Industrial, Kibbutz Beit-Haemek, Israel), 100 U/mL of penicillin (Gibco, Grand Island, NY,), and 100 pg/mL of streptomycin (Gibco, Grand Island, NY, in a humidified atmosphere of 5% CO_2_/95% air at 37°C [39].

### Ethics statement

All the experiments followed guidelines pertaining to experimental animal welfare and were permitted by the IUAUC of Mackay Memorial Hospital (MMH-A-S-100-44).

### Silica-core gold nanoshell preparation

Silica-core gold nanoshells (SGNs) were prepared following a procedure proposed in previous studies with several modifications [40]. Briefly, tetraethylorthosilicate (1.5 mL; Sigma, St. Louis, MO) was condensed in a mixture of 3 mL of 28% ammonia hydroxide (Sigma, St. Louis, MO, USA) and 50 mL of absolute ethanol at room temperature overnight to form silica nanoparticles. Subsequently, 50 μL of 3-aminopropyltrimethoxysilane (APTMS; Sigma, St. Louis, MO) was added into vigorously stirred 50 mL of silica nanoparticles, and the stirring continued for 2 h to facilitate surface amino functionalization. Precipitated products were washed twice with ethanol and stored at 4°C in 50 mL of ethanol before use. To prepare a gold colloid as a nucleated center for producing gold nanoshells, 2 mL of 1% HAuCl_4_ (27 mmol; Alfa Aesar, Ward Hill, MA) was quickly added into a stirred mixture of 45 mL of deionized H_2_O, 0.5 mL of 1 M NaOH (Sigma, St. Louis, MO), and 1 mL of THPC solution (12 μL of 80% THPC diluted in 1 mL of H_2_O; Sigma, St. Louis, MO); the stirring continued for 20 min to form the gold colloid. After filtration using a 0.2-μm filter, the gold colloid was aged for 2 weeks at 4°C. Subsequently, the aged gold colloid was seeded onto silica nanoparticles by mixing the prepared gold colloid with APTMS-functionalized silica nanoparticles at a volume ratio of 10:1. The mixture was incubated at 4°C overnight and washed twice with distilled water through centrifugation. Then, the gold colloid-seeded silica nanoparticles were resuspended in distilled water to the same volume as original gold colloid added. Finally, 1 mL of the gold colloid-seeded silica nanoparticles were added to 80 ml of stirred K-gold solution (25 mg of potassium carbonate; Sigma, St. Louis, MO) and 1 mL of 1% HAuCl_4_ in 100 mL of distilled water) followed by 0.8 mL of formaldehyde (Sigma, St. Louis, MO); the stirring continued for 20 min to produce the gold nanoshell on the silica core. The SGNs were washed twice with 10% Trehalose (Sigma, St. Louis, MO), centrifuged at 620 × g for 12 min, and subsequently stored at 4°C.

### Conjugation of antibodies on silica-core gold nanoshells

Conjugation of antibodies on SGNs was performed according to methods used in previous studies [15]. Briefly, 100 μg of mouse anti-human CD47 antibody or isotype control (eBioscience, San Diego, CA) were linked to OPSS-PEG-NHS (MW = 5000; Creative PEGWorks, Winston Salem, NC) through incubation with 1 mL of 6.67 × 10^−5^ M OPSS-PEG-NHS and 100 mM sodium bicarbonate (pH 8.5; Sigma, St. Louis, MO) at 4°C overnight. The OPSS-PEG-NHS-linked antibodies were mixed into solution containing SGNs (OD_800_ = 6) at a ratio of 1:9 and incubated at 4°C overnight. Methyl-PEG-thiol (mPEG-SH, MW = 5000; Creative PEGWorks, Winston Salem, NC) was subsequently added at a concentration of 50 μM and incubated for 30 min to pegylate the antibody-conjugated SGNs. Excessive mPEG-SH was removed by washing with 10% Trehalose and centrifugation at 620 × g for 12 min. Bare SGNs were pegylated using the same method. The pegylated SGNs (pSGNs) were resuspended in 10% Trehalose and stored at 4°C.

### Characterization of pegylated silica-core gold nanoshells

One milliliter of resuspended pSGNs was injected into a 1-cm light-path cuvette. The absorption spectrum of pSGNs was scanned using a UV-visible spectrophotometer. The morphology of the pSGNs was observed using a TEM. Ten microliters of pSGNs were loaded onto carbon-coated grids and placed on top of Watman paper to absorb excess solvent. The TEM images were recorded with a JEOL JEM-1200EXII operating at the bias voltage of 80 kV. To determine the particle concentration, the absorption of pSGNs was first adjusted to OD_800_ = 6 and subsequently serially diluted in distilled water. Next, the diluted pSGNs were mixed with 1.85 × 10^6^/mL of 3-μm latex beads (Sigma, St. Louis, MO) at a ratio of 1:1 and evenly separated on 0.1-μm filter membranes (Millipore, Tullagreen, Ireland) through suction. Next, the membranes were coated with platinum and observed using an SEM (s-3500N, Hitachi, Tokyo, Japan). The SEM images were analyzed using ImageJ software to count the ratio of the pSGNs and 3-μm latex beads.

### Image analysis of the binding between antibody-conjugated pegylated silica-core gold nanoshells and cancer cells

First, 5 × 10^4^ of ID8 cells or TOV21G cells were seeded into a 24-well plate and incubated at 37°C overnight, and 100 μL of anti-human CD47 antibody or isotope control conjugated pSGNs (OD_800_ = 6) were then added into the cultured cells. The cells were incubated overnight and washed twice in culture medium through gentle pipetting. The binding of the pSGNs and cells was observed using a reverse DIC microscope (640x; Zeiss, Oberkochen, Germany).

### Flow analysis

Fifty thousand TOV21G or ID8 cells resuspended in cell culture medium were mixed with 100 μL of anti-human CD47 antibody or isotype antibody conjugated pSGNs and stored at room temperature for 30 min. The cells were washed in cell culture medium and incubated with 100 μL of PE-conjugated anti-mouse IgG antibody (Jackson ImmunoResearch, Newmarket, UK) at room temperature for 20 min in the dark. The cells incubated with isotype antibodies were used as the control. After washing, the cells were analyzed using flow cytometry (Calibur, BD Bioscience, San Diego, CA).

### Temperature measurement of the near-infrared laser irradiated solution containing pegylated silica-core gold nanoshells

First, pSGNs (OD_800_ = 6) were diluted (1:3) in RPMI 1640 cell culture medium. One milliliter of diluted pSGNs was transferred to a 24-well plate and irradiated using a 2000-mW NIR laser at a wavelength of 808 nm (LaserTo, Hong Kong, China). The rising temperature was measured using a thermocouple (1305 Thermometer; TES, Taipei, Taiwan) every 30 s from 0 to 300 s during laser irradiation. In addition, pSGNs-free culture medium well was irradiated with an NIR laser as a control.

### Photothermal treatment of ovarian cancer cells *in vitro*

To evaluate the photothermal effects on cell survival, 1 × 10^5^ of ID8 cells were seeded into a 24-well plate and incubated overnight. Next, 1 mL of diluted pSGNs was added into the wells and incubated for 1 h at 37°C. The cells inside the well were irradiated with a 2000 mW NIR laser until the temperature increased to 42°C. The cells cultured in pSGNs-free medium were irradiated for the same duration. After NIR laser irradiation, the cells were washed twice in RPMI 1640 medium with 10% FBS and cultured overnight at 37°C in an incubator. Subsequently, these cells were double stained with Hoechst 33342 (5 μg/mL) and propidium iodide (5 μg/mL, Sigma, St. Louis, MO) for 5 min and observed using a fluorescence microscope. The viability of the cells was evaluated using an MTT assay.

Photothermal ablation of the cells with target-specific pSGNs *in vitro* was conducted using a procedure similar to those used in previous studies [41–43]. Briefly, 1 × 10^5^ of ID8 cells and 2 × 10^5^ of TOV21G cells were seeded in 24-well plates and grown until they were nearly confluent. The cells were then washed once with phosphate buffered saline, and the antibody-conjugated pSGNs at OD_800_ = 6 was mixed into the cell culture medium at a ratio of 1:10 and added to each well. After a 2-h incubation, the cells were washed twice in new medium to remove unbound nanoshells; next, each well was irradiated with a 2000-mW NIR laser for 1, 2, and 5 min. On the next day, all cells were stained with Hochest 33342 and propidium iodide and observed using a fluorescence microscope.

### Heat treatment of cancer cells *in vitro*

One hundred thousand ID8 cells were seeded into a 24-well plate and incubated overnight. The cells were incubated at room temperature for 20 min, further incubated at 35°C and 42°C for 10 min, and then transferred back into a 37-°C incubator. On the next day, the cell viability was analyzed using an MTT assay.

### Near-infrared laser penetration test

One hundred microliters of solution containing pSGNs (OD_800_ = 6) were added into a 96-well plate. A piece of abdomen wall dissected from an NOD/SCID mouse was placed on top of the culture well containing the solution of pSGNs. An NIR laser irradiated through the abdominal skin, and the thermocouple was placed into the pSGNs-solution-containing well to measure the temperature of the solution. The temperature curves of the solution containing pSGNs and the control solution containing distilled water that was irradiated with an NIR laser were recorded.

### *In vivo* intraperitoneal tissue injury detection

Thirty thousand ID8-luc cells were intraperitoneally injected into the peritoneums of the NOD/SCID mice. These tumor-bearing mice were intraperitoneally injected with 2 mL of solution containing pSGNs (OD_800_ = 1.5; for pSGNs-only and NIR + pSGNs groups) and 10% trehalose (for control and NIR-only groups). The mice were anesthetized with Zoletil (Vibac S.A., Carros, France; 25 mg/kg, intraperitoneal injection) and Xylazine (Bayer HealthCare, Levekusen, Germany; 10 mg/kg, i.p. injection), and the abdomen was swabbed with glycerol prior to laser irradiation. The mice receiving pSGNs underwent 808 nm of NIR laser irradiation in five areas for a total of 5 min on the abdomen at 3.2 W/cm^2^ using a 0.54-cm^2^ laser spot. On the next day, the ID8 cells inside the peritoneums of each group were lavaged and harvested. Red blood cells were depleted using an ACK Lysing buffer (Invitrogen, Carlsbad, CA) and the remaining cells were stained with annexin V and PI according to the manufacturer's instructions (Serotec, Oxford, UK). GFP-positive ID8 cells were specifically gated separately from other host cells and the results from the staining were analyzed using flow cytometry (Caliber, BD bioscience, San Diego, CA). To examine the damage of normal tissues, the liver, kidney, spleen, and intestine dissected from each group were fixed overnight in 10% formalin solution and then embedded in paraffin. Apoptosis of the normal tissues was determined through TUNEL staining (ApopTag Plus Peroxidase In Situ Apoptosis Detection Kit, Millipore, Tullagreen, Ireland), which was performed according to the manufacturer's instructions.

### Ovarian cancer models treated with pegylated silica-core gold nanoshell-mediated intraperitoneal hyperthermia

NOD/SCID mice were intraperitoneally administered 1 × 10^6^ of TOV21G-luc cells and 3 × 10^5^ of ID8-luc cells to form the tumor. Next, these tumor-bearing mice were intraperitoneally injected with 2 mL of pSGNs solution (OD_800_ = 1.5; for pSGNs only and NIR + pSGNs only groups, *N* = 5 each) or 10% trehalose (for control and NIR-only groups, *N* = 5 each). The mice were then anesthetized using Zoletil (25 mg/kg, i.p. injection) and Xylazine (10 mg/kg, i.p. injection). The mouse abdomen was swabbed with glycerol prior to laser irradiation. The NIR-only and NIR + pSGNs groups received 808-nm NIR laser irradiation in five different areas for total 5 min on the abdomen at 3.2 W/cm^2^ using a 0.54-cm^2^ laser spot. The NIR laser irradiation was repeated every 3 to 4 days, and pSGNs were supplemented before the second and fourth session of laser irradiation. The tumor growth was monitored on the basis of luminescence activity and determined using a noninvasive IVIS system (Xenogen, Grantham, UK). Tumor-targeting pSGNs were prepared through conjugation with anti-human CD47 antibodies (CD47-pSGNs). The CD47-conjugated pSGNs (OD_800_ = 0.625) and isotype-conjugated pSGNs (OD_800_ = 1.5) were intraperitoneally injected into TOV21G-bearing mice 7 days after tumor cell implantation (*N* = 5 for each group). The mice were irradiated and monitored as previously described.

### Statistics

All results were presented as the mean ± SE (standard error) from at least two independent experiments. Comparisons between various data points were performed using a Student's *t* test.

## SUPPLEMENTARY FIGURES



## References

[1] Jemal A, Bray F, Center MM, Ferlay J, Ward E, Forman D (2011). Global cancer statistics. CA: a cancer journal for clinicians.

[2] Bast RC, Hennessy B, Mills GB (2009). The biology of ovarian cancer: new opportunities for translation. Nature reviews Cancer.

[3] Badgwell D, Bast RC (2007). Early detection of ovarian cancer. Disease markers.

[4] Kobayashi E, Ueda Y, Matsuzaki S, Yokoyama T, Kimura T, Yoshino K (2012). Biomarkers for screening, diagnosis, and monitoring of ovarian cancer. Cancer epidemiology, biomarkers & prevention: a publication of the American Association for Cancer Research, cosponsored by the American Society of Preventive Oncology.

[5] Vasey PA, Herrstedt J, Jelic S, Force EGT (2005). ESMO Minimum Clinical Recommendations for diagnosis, treatment and follow-up of epithelial ovarian carcinoma. Annals of oncology : official journal of the European Society for Medical Oncology / ESMO.

[6] Kim A, Ueda Y, Naka T, Enomoto T (2012). Therapeutic strategies in epithelial ovarian cancer. Journal of experimental & clinical cancer research: CR.

[7] Lu Z, Wang J, Wientjes MG, Au JL (2010). Intraperitoneal therapy for peritoneal cancer. Future Oncol.

[8] Di Giorgio A, Naticchioni E, Biacchi D, Sibio S, Accarpio F, Rocco M (2008). Cytoreductive surgery (peritonectomy procedures) combined with hyperthermic intraperitoneal chemotherapy (HIPEC) in the treatment of diffuse peritoneal carcinomatosis from ovarian cancer. Cancer.

[9] Helm CW (2009). The role of hyperthermic intraperitoneal chemotherapy (HIPEC) in ovarian cancer. The oncologist.

[10] Song CW (1984). Effect of local hyperthermia on blood flow and microenvironment: a review. Cancer research.

[11] van Ruth S, Verwaal VJ, Hart AA, van Slooten GW, Zoetmulder FA (2003). Heat penetration in locally applied hyperthermia in the abdomen during intra-operative hyperthermic intraperitoneal chemotherapy. Anticancer research.

[12] Chatterjee DK, Diagaradjane P, Krishnan S (2011). Nanoparticle-mediated hyperthermia in cancer therapy. Therapeutic delivery.

[13] Oldenburg S, Averitt R, Westcott S, Halas N (1998). Nanoengineering of optical resonances. Chemical Physics Letters.

[14] Weissleder R (2001). A clearer vision for *in vivo* imaging. Nature biotechnology.

[15] Carpin LB, Bickford LR, Agollah G, Yu TK, Schiff R, Li Y (2011). Immunoconjugated gold nanoshell-mediated photothermal ablation of trastuzumab-resistant breast cancer cells. Breast cancer research and treatment.

[16] Day ES, Thompson PA, Zhang L, Lewinski NA, Ahmed N, Drezek RA (2011). Nanoshell-mediated photothermal therapy improves survival in a murine glioma model. Journal of neuro-oncology.

[17] Hirsch LR, Stafford R, Bankson J, Sershen S, Rivera B, Price R (2003). Nanoshell-mediated near-infrared thermal therapy of tumors under magnetic resonance guidance. Proceedings of the National Academy of Sciences.

[18] O'Neal DP, Hirsch LR, Halas NJ, Payne JD, West JL (2004). Photo-thermal tumor ablation in mice using near infrared-absorbing nanoparticles. Cancer letters.

[19] Cheng F-Y, Chen C-T, Yeh C-S (2009). Comparative efficiencies of photothermal destruction of malignant cells using antibody-coated silica@ Au nanoshells, hollow Au/Ag nanospheres and Au nanorods. Nanotechnology.

[20] Poels LG, Peters D, van Megen Y, Vooijs GP, Verheyen RN, Willemen A (1986). Monoclonal antibody against human ovarian tumor-associated antigens. Journal of the National Cancer Institute.

[21] Campbell IG, Freemont PS, Foulkes W, Trowsdale J (1992). An ovarian tumor marker with homology to vaccinia virus contains an IgV-like region and multiple transmembrane domains. Cancer research.

[22] Wu C-C, Chuang Y-T, Hsu Y-T, Huang J-T, Wu T-C, Hung C-F (2013). Intra-Peritoneal Hyperthermia Combining α-Galactosylceramide in the Treatment of Ovarian Cancer. PloS one.

[23] Abdo A, Ersen A, Sahin M (2013). Near-infrared light penetration profile in the rodent brain. Journal of biomedical optics.

[24] Storm FK, Harrison WH, Elliott RS, Morton DL (1979). Normal tissue and solid tumor effects of hyperthermia in animal models and clinical trials. Cancer research.

[25] Chao MP, Alizadeh AA, Tang C, Jan M, Weissman-Tsukamoto R, Zhao F (2011). Therapeutic antibody targeting of CD47 eliminates human acute lymphoblastic leukemia. Cancer research.

[26] Chao MP, Alizadeh AA, Tang C, Myklebust JH, Varghese B, Gill S (2010). Anti-CD47 antibody synergizes with rituximab to promote phagocytosis and eradicate non-Hodgkin lymphoma. Cell.

[27] Manna PP, Frazier WA (2004). CD47 mediates killing of breast tumor cells via Gi-dependent inhibition of protein kinase A. Cancer research.

[28] Chan KS, Espinosa I, Chao M, Wong D, Ailles L, Diehn M (2009). Identification, molecular characterization, clinical prognosis, and therapeutic targeting of human bladder tumor-initiating cells. Proceedings of the National Academy of Sciences of the United States of America.

[29] Yang J, Lee J, Kang J, Oh SJ, Ko HJ, Son JH (2009). Smart Drug-Loaded Polymer Gold Nanoshells for Systemic and Localized Therapy of Human Epithelial Cancer. Advanced Materials.

[30] Cardinal J, Klune JR, Chory E, Jeyabalan G, Kanzius JS, Nalesnik M (2008). Noninvasive radiofrequency ablation of cancer targeted by gold nanoparticles. Surgery.

[31] Curley SA, Cherukuri P, Briggs K, Patra CR, Upton M, Dolson E (2008). Noninvasive radiofrequency field-induced hyperthermic cytotoxicity in human cancer cells using cetuximab-targeted gold nanoparticles. Journal of experimental therapeutics & oncology.

[32] Bastus NG, Kogan MJ, Amigo R, Grillo-Bosch D, Araya E, Turiel A (2007). Gold nanoparticles for selective and remote heating of β-amyloid protein aggregates. Materials Science and Engineering: C.

[33] Araya E, Olmedo I, Bastus NG, Guerrero S, Puntes VF, Giralt E (2008). Gold nanoparticles and microwave irradiation inhibit beta-amyloid amyloidogenesis. Nanoscale research letters.

[34] Tang L, Yang X, Yin Q, Cai K, Wang H, Chaudhury I (2014). Investigating the optimal size of anticancer nanomedicine. Proceedings of the National Academy of Sciences.

[35] Hirsch LR, Stafford RJ, Bankson JA, Sershen SR, Rivera B, Price RE (2003). Nanoshell-mediated near-infrared thermal therapy of tumors under magnetic resonance guidance. Proceedings of the National Academy of Sciences of the United States of America.

[36] Loo C, Lin A, Hirsch L, Lee MH, Barton J, Halas N (2004). Nanoshell-enabled photonics-based imaging and therapy of cancer. Technology in cancer research & treatment.

[37] Loo C, Lowery A, Halas N, West J, Drezek R (2005). Immunotargeted nanoshells for integrated cancer imaging and therapy. Nano letters.

[38] James W, Hirsch L, West J, O'Neal P, Payne J (2007). Application of INAA to the build-up and clearance of gold nanoshells in clinical studies in mice. Journal of Radioanalytical and Nuclear Chemistry.

[39] Chang CL, Hsu YT, Wu CC, Lai YZ, Wang C, Yang YC (2013). Dose-dense chemotherapy improves mechanisms of antitumor immune response. Cancer research.

[40] Pham T, Jackson JB, Halas NJ, Lee TR (2002). Preparation and characterization of gold nanoshells coated with self-assembled monolayers. Langmuir: the ACS journal of surfaces and colloids.

[41] Loo C, Lowery A, Halas N, West J, Drezek R (2005). Immunotargeted nanoshells for integrated cancer imaging and therapy. Nano letters.

[42] Bernardi RJ, Lowery AR, Thompson PA, Blaney SM, West JL (2008). Immunonanoshells for targeted photothermal ablation in medulloblastoma and glioma: an *in vitro* evaluation using human cell lines. Journal of neuro-oncology.

[43] Lowery AR, Gobin AM, Day ES, Halas NJ, West JL (2006). Immunonanoshells for targeted photothermal ablation of tumor cells. International journal of nanomedicine.

