# Supplementary material for: Nanoparticle-induced intraperitoneal hyperthermia and targeted photoablation in treating ovarian cancer
Source: Oncotarget. 2015 Aug 3;6(29):26861–75. doi: 10.18632/oncotarget.4766 (PMC4694958; doi:10.18632/oncotarget.4766)
Supplement: Supplementary file 1 [file oncotarget-06-26861-s001.pdf]

## SUPPLEMENTARY FIGURES

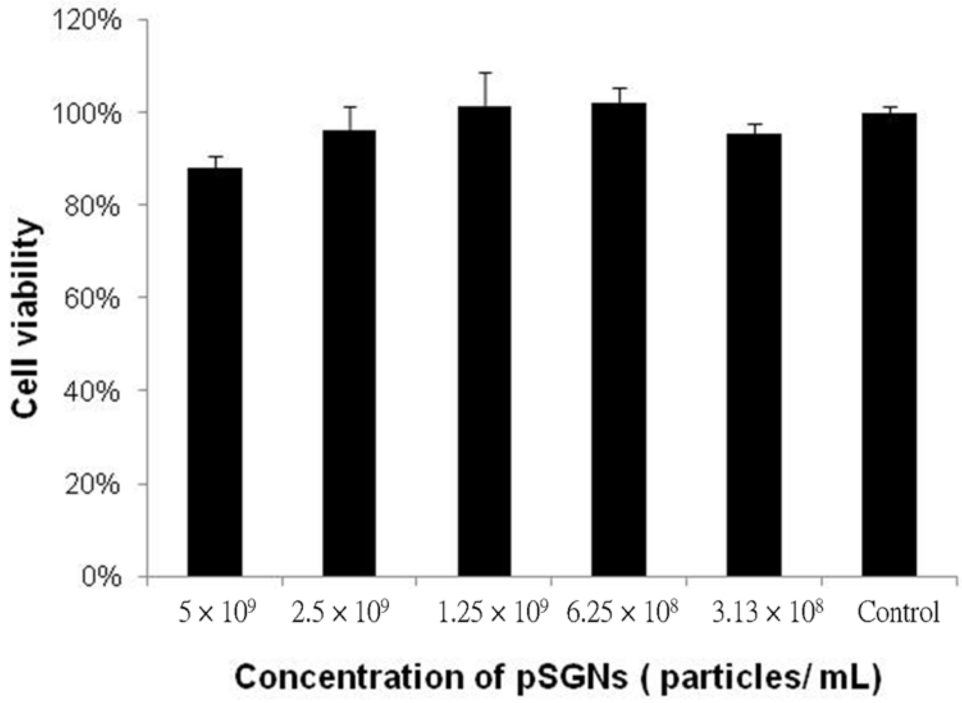


**Supplementary Figure S1: Cytotoxicity assay of gold nanoshell *in vitro*.** One hundred thousand ID8 cells were seeded into a well in a 6-well plate and cultured overnight. The culture medium was discarded and replaced with 1 mL of medium containing various concentrations of pSGNs. Cells without treatment were used as control. The cells were cultured for 48 h, and their survival was analyzed using an MTT assay. No significant decrease of the survival rate was observed in the cells cocultured with each concentration of pSGNs.

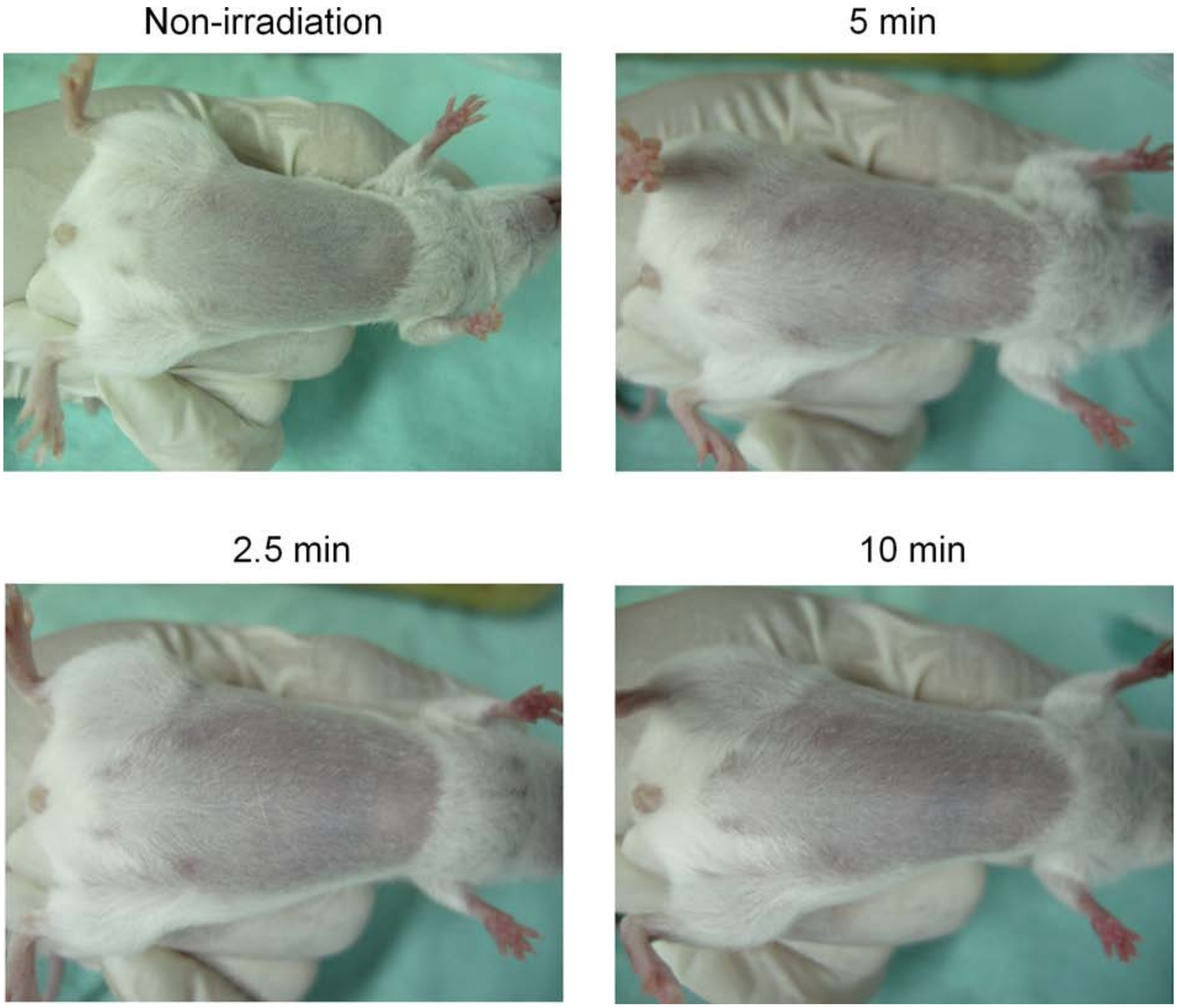


**Supplementary Figure S2: Evaluation of the abdominal skin damage after irradiation with various doses of NIR laser.** The abdomens of the test NOD-SCID mice were shaved and glued with glycerol. The mice received NIR irradiation in five areas for a total of 2.5, 5, and 10 min. Mice that did not receive NIR irradiation were used as control. After 24 h, no skin damage was observed on the abdomen of the mice that received NIR irradiation.

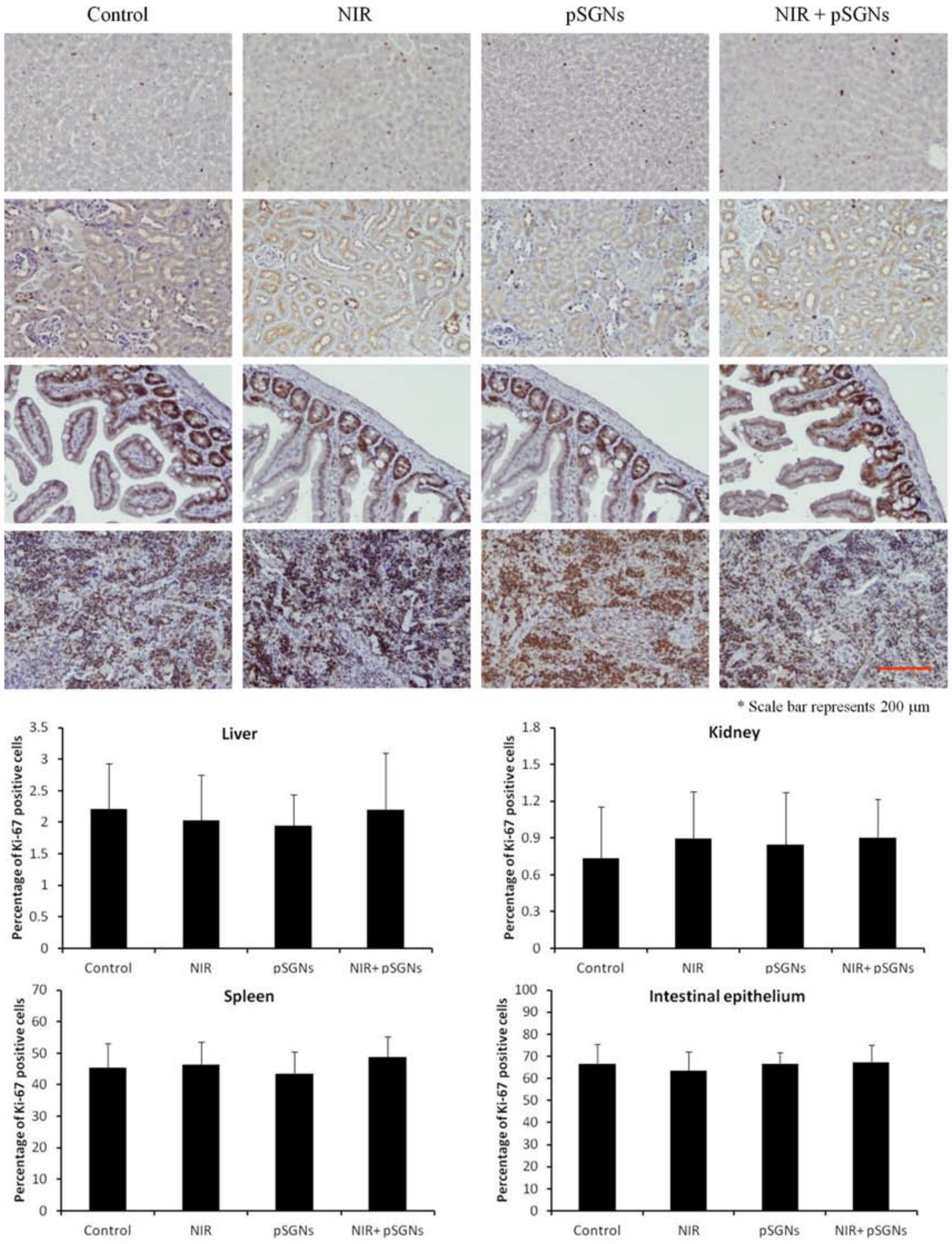


**Supplementary Figure S3: Ki-67 staining of intraperitoneal organ in mice intraperitoneally injected with pSGNs following NIR irradiation.** The NOD-SCID mice were divided into four groups and intraperitoneally injected with 2 mL of pSGNs ($OD_{800}$ = 1.5) or 10% trehalose and irradiated with an 808-nm NIR laser (3.2 W/$cm^2$) in five areas on the abdomen for a total of 5 min. One day after the final NIR irradiation, the liver, kidney, spleen, and intestine from the mice of each group were dissected and fixed in 10% formalin. The cell proliferation pattern of each organ was evaluated using immunohistochemistry with anti-mouse Ki-67 antibody staining (Thermo Scientific), and the percentage of Ki-67 positive cells was counted using ImageJ software. The size of the scale bar was 200 µm. The results showed that photothermal therapy did not affect cell proliferation in normal intraperitoneal tissues *in vivo*. The error bar represents the standard error.

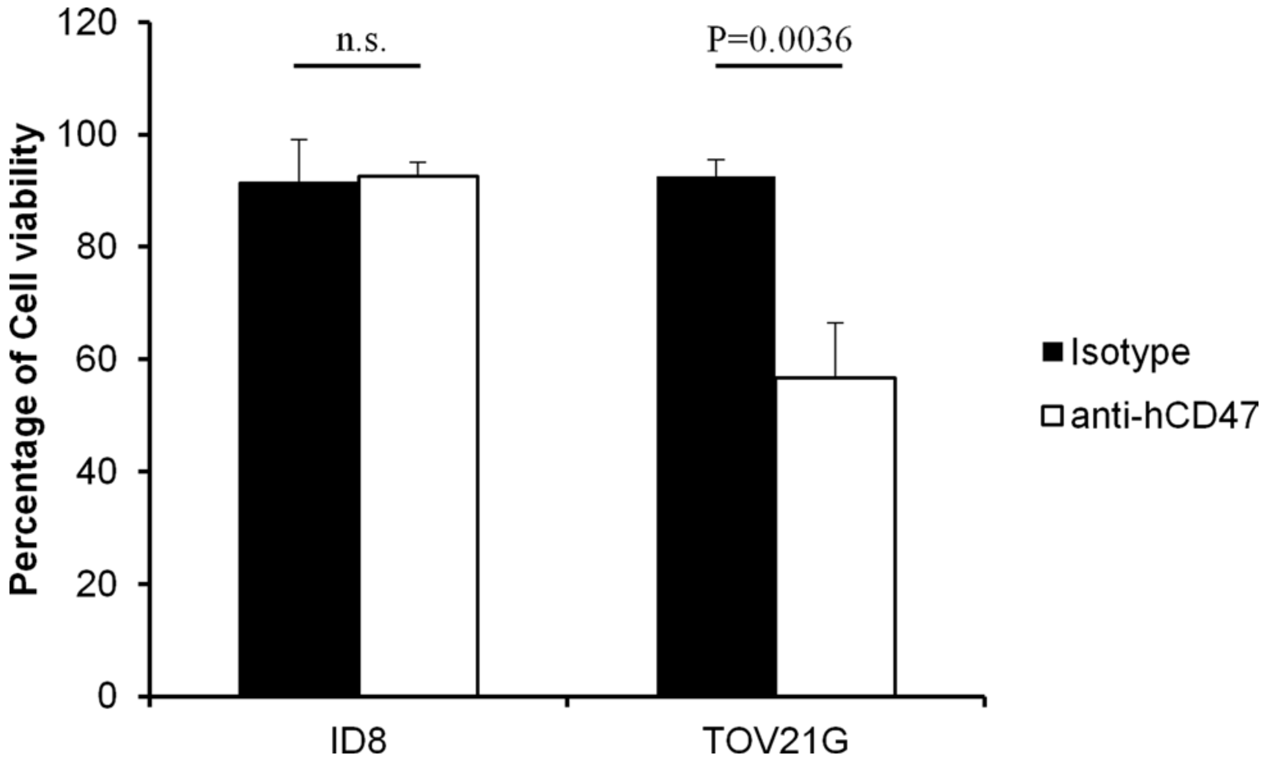


**Supplementary Figure S4: Photothermal cytotoxicity of hCD47-pSGNs.** A total of 1 × $10^4$ ID8 and 2 × $10^4$ TOV21G cells were seeded into 96-well plates and cultured overnight at 37°C. The antibody-conjugated pSGNs ($OD_{800}$ = 3) were mixed with cell culture medium at a ratio of 1:5, added into the ID8 or TOV21G cell culture well, and cultured for 2 h. The cells were washed twice in fresh medium and then irradiated using a 2000-mW NIR laser on each well for 5 min. All cells were cultured for 24 h and the cell viability was determined using an MTT assay. The results demonstrated that anti-human CD47 antibody-conjugated pSGNs can specifically bind to human TOV21G ovarian cancer cells, but not to mouse cells, and cause cell death through photothermal effects. The error bar in each chart represents the standard error.

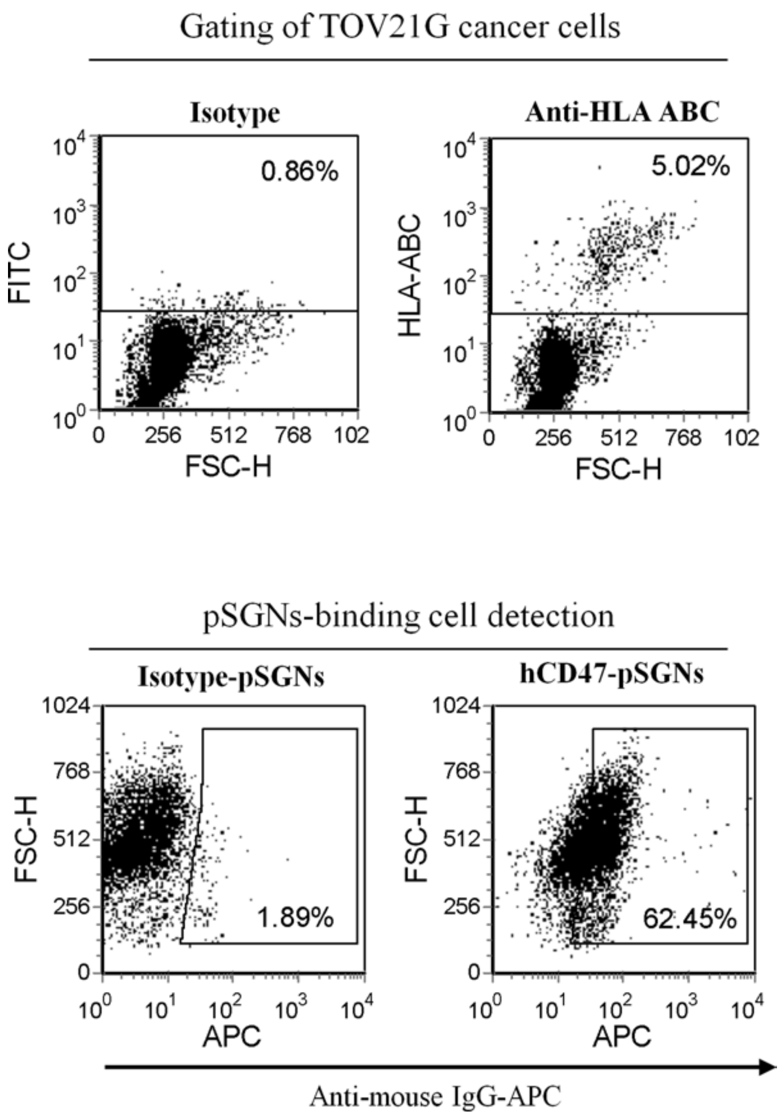


**Supplementary Figure S5: Targeting of hCD47-pSGNs *in vivo*.** Three million human TOV21G cells were i.p. implanted into NOD/SCID mic. After three days, mice were given i.p. 2 mL of mouse IgG1-pSGNs and hCD47-pSGNs ($OD_{800}$ = 0.625). Mice were then sacrificed and i.p. lavaged to collect the cells within the peritoneal cavity. The harvested cells were sequentially stained with anti-mouse IgG-APC and anti-human HLA-ABC-FITC. The HLA-ABC positive cancer cells were first gated (to filter out mouse cells) and hCD47-pSGNs associated cells were analyzed using flow cytometry for APC positive cells, indicating recognition of mouse antibody-pSGNs. The results demonstrated that the hCD47-pSGNs, but not the isotype antibody-conjugated pSGNs, can bind to human TOV21G cells *in vivo*.

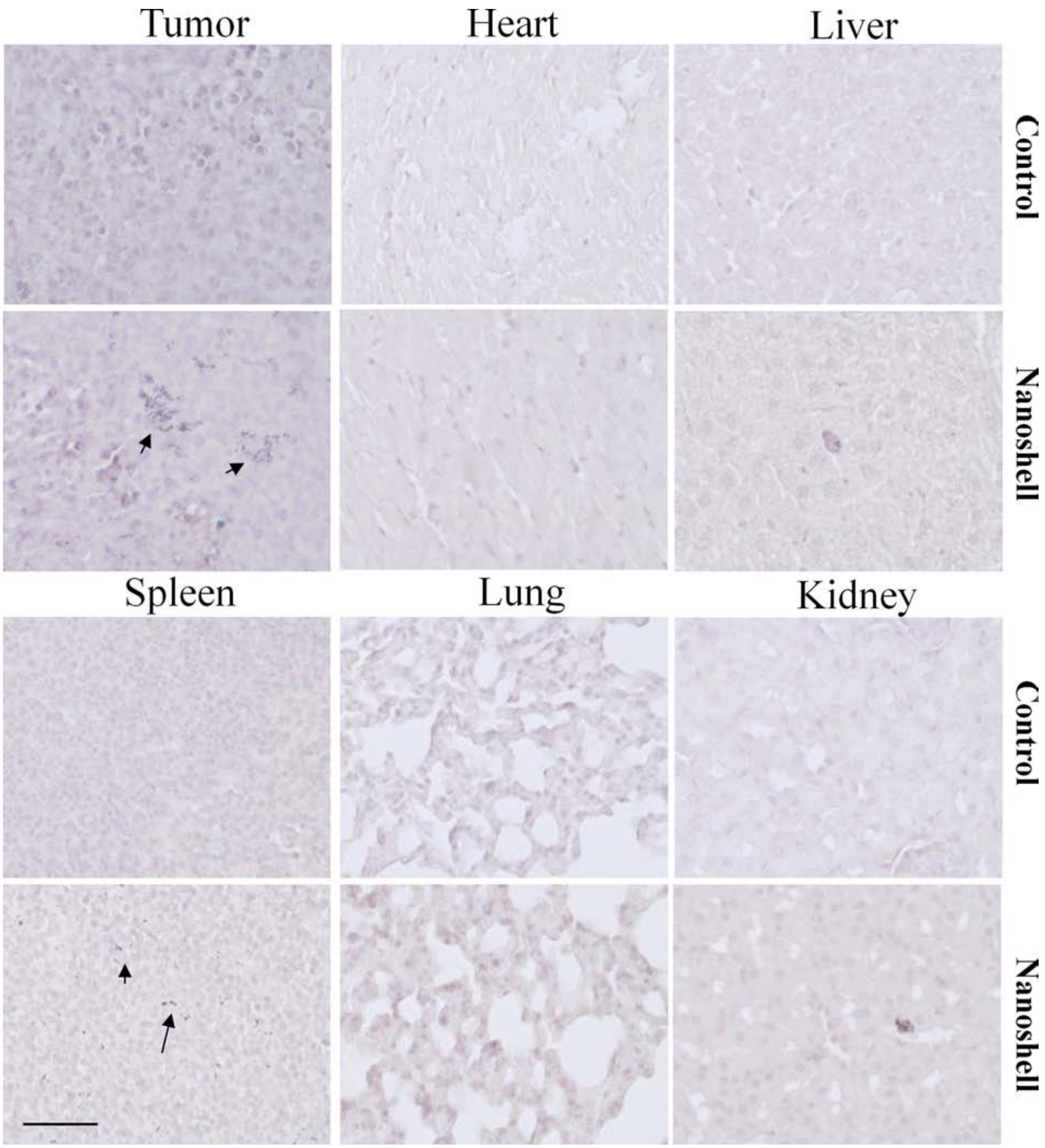


**Supplementary Figure S6: Biodistribution of silica-core gold nanoshells in tumor-bearing mice.** The B6C3F1 mice were subcutaneously injected with 1 × $10^6$ HM-1 cells. After 7 days, 200 μL of pegylated silica-core gold nanoshells ($OD_{800}$ = 6) were injected into each mouse through the tail vein. The mice were sacrificed after 2 days; the organs were fixed using formaldehyde and then embedded in paraffin. Gold nanoshells in various organs were labeled using silver stains and observed using light microscopy. The arrows indicate the stains of gold nanoparticles.
